# Exploring the Chemical Composition and Antimicrobial Activity of Extracts from the Roots and Aboveground Parts of *Limonium gmelini*

**DOI:** 10.3390/molecules30143024

**Published:** 2025-07-18

**Authors:** Dariya Kassymova, Francesco Cairone, Donatella Ambroselli, Rosa Lanzetta, Bruno Casciaro, Aizhan Zhussupova, Deborah Quaglio, Angela Casillo, Galiya E. Zhusupova, Maria Michela Corsaro, Bruno Botta, Silvia Cammarone, Maria Luisa Mangoni, Cinzia Ingallina, Francesca Ghirga

**Affiliations:** 1 Department of Chemistry and Technology of Organic Substances, Natural Compounds and Polymers, Faculty of Chemistry and Chemical Technology, NPJSC Al-Farabi Kazakh National University, Al-Farabi Ave. 71, Almaty 050040, Kazakhstan; darik459@gmail.com; 2 Department of Chemistry and Technology of Drugs, Sapienza University of Rome, Piazzale Aldo Moro 5, 00185 Rome, Italy; francesco.cairone@uniroma1.it (F.C.); donatella.ambroselli@uniroma1.it (D.A.); deborah.quaglio@uniroma1.it (D.Q.); bruno.botta@uniroma1.it (B.B.); cinzia.ingallina@uniroma1.it (C.I.); francesca.ghirga@uniroma1.it (F.G.); 3 Department of Chemical Sciences, Complesso Universitario di Monte S. Angelo, University of Napoli Federico II, Via Cintia 4, 80126 Napoli, Italy; rosa.lanzetta@unina.it (R.L.); angela.casillo@unina.it (A.C.); mariamichela.corsaro@unina.it (M.M.C.); 4 Laboratory Affiliated to Istituto Pasteur Italia-Fondazione Cenci Bolognetti, Department of Biochemical Sciences, Sapienza University of Rome, 00185 Rome, Italy; bruno.casciaro@uniroma1.it (B.C.); marialuisa.mangoni@uniroma1.it (M.L.M.); 5 Department of Molecular Biology and Genetics, Faculty of Biology and Biotechnology, NPJSC Al-Farabi Kazakh National University, Al-Farabi Ave. 71, Almaty 050040, Kazakhstan; aizhan.zhussupova@kaznu.edu.kz

**Keywords:** *Limonium gmelini*, polysaccharides, polyphenols, monosaccharide composition, monosaccharide linkages, antibacterial activity

## Abstract

*Limonium gmelini* (*Willd.*) Kuntze, a plant widely used in traditional medicine, has garnered increasing attention for its diverse pharmacological activities, including anti-inflammatory, hepatoprotective, antioxidant, and antimicrobial effects. This study aimed to explore the chemical composition and biological activities of polysaccharides and polyphenolic compounds extracted from both the roots and aboveground parts of *Limonium gmelini*. Several methods of extraction, including ultrasound-assisted extraction (UAE), conventional maceration (CM), and supercritical fluid extraction (SFE), were employed to obtain bioactive fractions. Chemical profiling, primarily represented by monosaccharides and polyphenolic compounds, was characterized and analyzed using proton nuclear magnetic resonance spectroscopy (^1^H-NMR) and gas chromatography-mass spectrometry (GC-MS) techniques. While polyphenol-rich fractions exhibited significant antibacterial activity, particularly against *Staphylococcus epidermidis*, polysaccharide-rich aqueous fractions showed minimal antibacterial activity. Among the methods, CM and UAE yielded higher polyphenol content, whereas SFE provided more selective extractions. Notably, methanolic SPE fractions derived from the roots were especially enriched in active polyphenols such as gallic acid, myricetin, and naringenin, and they exhibited the highest antibacterial activity against Staphylococcus epidermidis. In contrast, extracts from the aboveground parts showed more moderate activity and a partially different chemical profile. These findings underscore the importance of plant part selection and support the targeted use of root-derived polyphenol-enriched fractions from *L. gmelini* as promising candidates for the development of natural antibacterial agents. Further investigation is needed to isolate and validate the most active constituents for potential therapeutic applications.

## 1. Introduction

*Limonium gmelini* (*Willd.*) Kuntze (Siberian statice), a resilient perennial species of the Plumbaginaceae family, has shown remarkable potential for medical and pharmaceutical applications due to its rich phytochemical profile, which includes both polysaccharides and polyphenolic compounds. Native to saline soil and coastal regions across Eurasia, *L. gmelini* is adapted to thrive under various environmental stresses, an attribute that may contribute to the high concentration of bioactive compounds with potential therapeutic benefits. Traditionally utilized for its wound-healing, anti-inflammatory, and antioxidant properties, this plant has been investigated in recent years for its antimicrobial effects, offering a natural solution to combat antibiotic-resistant pathogens [1,2].

Among the active constituents of *L. gmelini*, polysaccharides and polyphenols play critical roles in its therapeutic potential [2,3,4,5,6,7]. Tendentially, the polysaccharidic compounds of *L. gmelini* are noted for their ability to disrupt microbial cell walls and biofilms, making it potent against pathogens, especially those associated with chronic infections [8]. The antimicrobial effects of these compounds, however, are highly influenced by the method of extraction, which affects both the yield and structural integrity of the polysaccharides [8,9]. Instead, polyphenolic compounds—including myricetin, quercitrin, and methyl gallate—further contribute to its pharmacological profile. Polyphenols, recognized for their antioxidant, anti-inflammatory, and antimicrobial properties, are present in both the aerial and root tissues of the plant. These compounds demonstrate significant free radical scavenging activity, thereby contributing to the attenuation of oxidative stress—a key factor implicated in the pathogenesis of chronic conditions such as cardiovascular and neurodegenerative diseases. Furthermore, polyphenols exert direct antimicrobial effects and can act synergistically with polysaccharides, resulting in enhanced inhibitory action against a wide range of pathogenic microorganisms [6].

Moreover, in traditional medicine, *L. gmelini* has been highly regarded for its wound-healing capabilities [10]. Noteworthy, the roots of *L. gmelini* were introduced into the State Pharmacopoeia of the Republic of Kazakhstan in 2009, with a monograph on its aboveground parts added in 2014. Medicinal products based on *L. gmelini*, unified under the brand name “Limonidin,” have been developed, including tinctures, ointments, and syrups [11]. Additionally, some studies reported that the ethyl acetate plant fractions enriched with quercitrin and myricetin showed strong antibacterial activity against *Staphylococcus (S.) aureus* [4,6]. Further, most of the available studies have been conducted on cell lines and animal models, particularly focusing on the antioxidant, anti-inflammatory, hepatoprotective, and antidiabetic activities of the plant [8,12].

Although *Limonium gmelini* has been previously studied for its general pharmacological effects, there is a lack of detailed investigations comparing extraction techniques and their influence on the chemical profile and bioactivity of different plant parts. The integration of advanced analytical techniques such as GC-MS and ^1^H-NMR with biological screening provides a novel and comprehensive insight into the relationship between extraction method, chemical composition, and antimicrobial activity. This approach lays the groundwork for a more targeted exploitation of this species in pharmaceutical applications.

Accordingly, this research aims to systematically evaluate the influence of different extraction methods on the yield and bioactivity of *L. gmelini* extracts, focusing not only on polysaccharides but also on secondary metabolites, such as polyphenols. Three extraction methods were selected: conventional maceration (CM), ultrasound-assisted extraction (UAE), and supercritical fluid extraction (SFE). CM is widely used due to its simplicity and broad applicability, although it typically requires longer times and higher solvent volumes. UAE, based on acoustic cavitation, offers improved efficiency and compound preservation, while SFE, using supercritical CO_2_ with co-solvents, provides a selective and environmentally friendly alternative. This methodological framework enables a direct comparison between traditional and green methods in terms of efficiency, selectivity, and sustainability [13]. The study compares extracts obtained from the roots and aboveground parts of the plant, allowing for the assessment of tissue-specific differences in compound composition and activity. By integrating extraction yield, phytochemical profiling (GC-MS, ^1^H-NMR), and in vitro antibacterial assays, we aim to identify the conditions that maximize the recovery of bioactive constituents, with particular attention to those involved in antimicrobial potential. 

Given the urgent need for new antimicrobial agents in the face of rising multidrug resistance, this study provides a foundation for evaluating *L. gmelini* fractions enriched in biologically active compounds as potential candidates for antimicrobial development. Such fractions, particularly those effective against Gram-positive/Gram-negative pathogens and capable of disrupting biofilms, may be of interest both as direct therapeutic candidates and as starting points for further investigation. Additional studies will be required to isolate, characterize, and validate the most active constituents for future pharmaceutical applications, especially in the context of rising antibiotic resistance in clinical and agricultural settings.

## 2. Results and Discussion

### 2.1. Comparative Analysis of the Methods for Obtaining Plant Extracts from the Roots and Aboveground Parts of L. gmelini

The roots and aboveground parts of *L. gmelini* were collected and botanically identified by the Institute of Botany and Phytointroduction (Almaty, Kazakhstan), while further investigations were carried out at Sapienza University of Rome (Italy).

**Figure 1:**
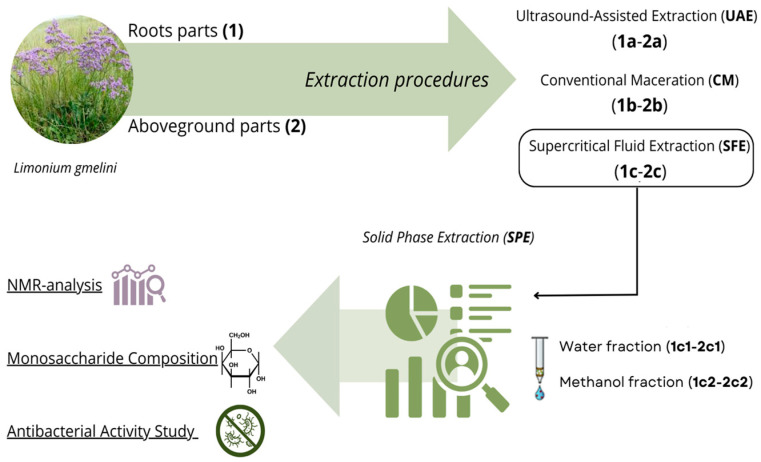
Schematic of extraction, purification, and analytical methods for *L. gmelini* Extracts. (Canva (web-based design platform), version as of March 2023, Canva Pty Ltd., Sydney, Australia).

In total, six extracts were prepared to assess these extraction methods, as illustrated by Figure 1: 1a, 1b, and 1c, derived from the roots using ultrasound-assisted extraction (UAE), conventional maceration (CM), and supercritical fluid extraction (SFE), respectively; and 2a, 2b and 2c, derived from the aboveground parts using the same techniques (UAE, CM, and SFE). SFE extracts were further purified by solid-phase extraction (SPE), yielding aqueous (1c1, 2c1) and methanolic (1c2, 2c2) fractions to improve compound selectivity and characterization.

**Figure 2:**
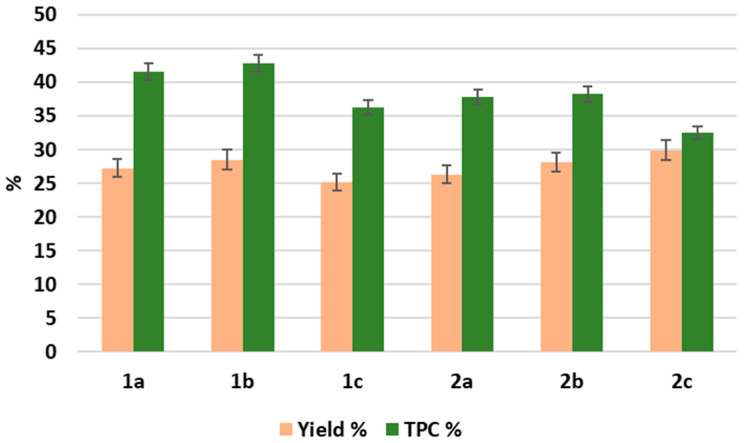
Comparative analysis of the methods for obtaining extracts from *L. gmelini*.

We specifically aimed to explore the extraction yields and the composition of polysaccharides and polyphenols, which represent key bioactive compounds with significant anti-inflammatory, immunomodulatory, and oncological prevention properties, among other therapeutic effects [14,15,16,17]. Their activity is modulated by structural features such as glycosidic linkages, branching, and molecular weight in polysaccharides, or the oxidation state and substitution pattern in polyphenols [18,19]. The extraction yields (%) and total polyphenol content (TPC) were recorded for each method, with results summarized in Figure 2.

The conventional extraction technique, maceration (CM), has been traditionally employed in both research and industrial settings to obtain a variety of compounds from different types of matrices. This method involves soaking plant material in a solvent to facilitate the release of bioactive compounds, typically requires a prolonged extraction time, and results in relatively high solvent consumption [20,21]. Despite these drawbacks, CM remains widely used due to its simplicity and effectiveness in extracting a broad range of compounds. In this study, CM resulted in high yields of 28.5% from the roots and 28.1% from the aboveground parts, with a total polyphenol content of 42.8% and 38.2%, respectively. The extracts obtained through CM were brown in color and crystalline in texture, indicating the successful extraction of the polyphenolic compounds [22]. Despite its effectiveness, CM is limited by its time- and resource-intensive nature, which often makes it less suitable for sustainable or high-throughput applications [20,21]. Given these limitations, to explore potentially more efficient and sustainable methods, we also applied non-conventional techniques, UAE and SFE, as alternative green analytical techniques that offer potential efficiency improvements while aligning with a more eco-friendly approach. These methods, designed to enhance extraction efficiency and reduce environmental impact, provided different results in terms of yield and polyphenol content. Specifically, UAE employs high-frequency sound waves (20 kHz to 100 MHz) to enhance mass transport through cavitation, disrupting plant cell walls and facilitating the release of extractable compounds [23,24]. This method provided brown powders similar to CM, but resulted in slightly lower extraction yields, with 27.3% from the roots and 26.3% from the aboveground parts and a TPC of 41.5% and 37.8%, respectively. This suggests that UAE is more effective in maintaining polyphenol extraction but has a slight reduction in yield when compared to CM.

On the other hand, SFE, which utilizes supercritical CO_2_ as a solvent, is considered eco-friendly and more sustainable than traditional solvent-based ways of extraction [25]. This method relies on a non-toxic, non-flammable, and easily recyclable material, making it an ideal option for minimizing environmental impact while achieving efficient extraction. While SFE is more selective for specific compounds, particularly for macromolecules, it initially yielded lower extraction efficiencies due to the poor solubility of polar compounds in pure CO_2_. However, the addition of ethanol and water as co-solvents significantly improved recovery. Specifically, the extraction yields from the roots and aboveground parts were 25.2% and 29.9%, respectively. The polyphenol content was 36.3% for the roots and 32.5% for the aboveground parts, approximately 6% lower than the same variables for UAE and CM extracts. These values are consistent with the higher selectivity of SFE for extracting polysaccharides and/or other macromolecules (e.g., proteins, lipids) rather than polyphenols.

Therefore, SFE extracts (1c and 2c) were further purified using solid-phase extraction (SPE). This step was implemented to refine the extract and isolate specific fractions. The purification process yielded two fractions from the roots: an aqueous fraction (1c1) and a methanolic fraction (1c2) with yields of 56.4% and 18.5%, respectively. Similarly, from the aboveground parts, two fractions were obtained: an aqueous fraction (2c1) and a methanolic fraction (2c2) with yields of 67.7% and 20.9%, respectively. Overall, among the methods employed, UAE, CM, and SFE yielded comparable amounts of bioactive compounds, with slightly higher yields observed for UAE and CM. However, SFE exhibited greater selectivity, as revealed by the differential composition of the SPE extracts. This result aligns with previous studies suggesting that UAE facilitates the breakdown of plant cell walls, thereby enhancing the release of secondary metabolites [12], while SFE enables a more targeted extraction of specific bioactive components. These findings emphasize the importance of selecting an appropriate extraction method with intended applications, balancing overall yield (UAE, CM) against compound-specific selectivity (SFE) [26,27]. In summary, both CM and UAE showed higher extraction yields and total polyphenol content. In contrast, SFE provided greater selectivity, particularly when followed by SPE purification. These differences suggest that the choice of extraction method should be based on the intended application, prioritizing either compound yield (CM, UAE) or targeted isolation (SFE).

### 2.2. Monosaccharide Composition

The dried extracts were dissolved in deionized water, and the polysaccharide fractions were obtained after precipitation with ethanol. The monosaccharide composition was obtained after derivatization as acetylated methyl glycosides (AMG). Furthermore, the linkage positions were determined by analyzing partially methylated alditol acetates (PMAAs) [28]. The obtained derivatives were analyzed using GC-MS and identified by retention time and fragmentation patterns compared to standards. The results of the monosaccharide composition are summarized in Table 1.

The results presented in Table 1 suggest that all the extracts obtained from the roots and aboveground parts of *L. gmelini* contained rhamnose (Rha), arabinose (Ara), glucuronic acid (GlA), mannose (Man), galactose (Gal), and glucose (Glc). Only the aboveground part extracts included xylose (Xyl) and galacturonic acid (GalA), indicating tissue-specific variation in carbohydrate expression.

**Figure 3:**
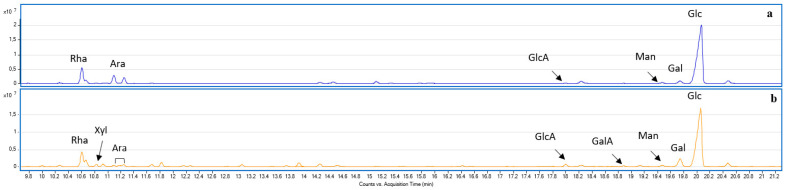
Chromatogram of AMG extracts related to 1b (**a**) and 2b (**b**) samples.

Figure 3 shows an example of GC-MS chromatograms of AMG from SFE-derived extracts of the roots (1b) and aboveground parts (2b), confirming the presence of a diverse range of monosaccharides in both plant tissues.

The chromatograms confirmed the detection of key monosaccharides, detailed in Table 1. The GC-MS analysis of PMAAs revealed the predominant linkage positions of monosaccharides in the polysaccharides isolated from the roots (1b) and aboveground parts of *L. gmelini* (2b), shown in Table 2.

The data revealed a significant presence of terminal non-reducing end arabinose and rhamnose in extracts from the roots and aboveground parts of *L. gmelini*.

In addition, the aboveground part extracts contained terminal xylose, while 3,4-linked rhamnose was consistently observed in both extracts, indicating more complex branching.

These findings suggest a complex carbohydrate backbone with specific branching patterns, highlighting the structural diversity of the polysaccharides. The presence of terminal arabinose, rhamnose, and xylose residues, along with 3,4-linked rhamnose, indicates a structurally complex and branched polysaccharide architecture. Such configurations have been associated with enhanced immunomodulatory properties in plant-derived polysaccharides [29]. Finally, since glucose did not appear in the PMAAs chromatogram, the possibility that the residue was a free monosaccharide was evaluated. After the acetylation of the extracts from the roots (1b) and aboveground parts (2b), a peracetylated glucose was identified by GC-MS, thus confirming the previous hypothesis. Overall, the monosaccharide analysis revealed a diverse composition, with rhamnose, arabinose, glucuronic acid, galacturonic acid, mannose, galactose, and glucose identified in varying proportions. Notably, xylose was present exclusively in the aboveground parts, suggesting potential structural differences in polysaccharide composition between the parts of the plant. The presence of these monosaccharides is associated with potential immunomodulatory and antioxidant properties of *L. gmelini* polysaccharides, as similar compositions have been linked to bioactive effects in other plant-derived polysaccharides [30,31,32,33,34].

### 2.3. Study of the Antibacterial Activity of L. gmelini Plant Extracts

The antibacterial activity of the studied plant extracts was assessed in vitro by evaluating their ability to inhibit bacterial growth using the inhibition zone assay—a diffusion method of growth inhibition on solid nutrient agar medium [35].

**Figure 4:**
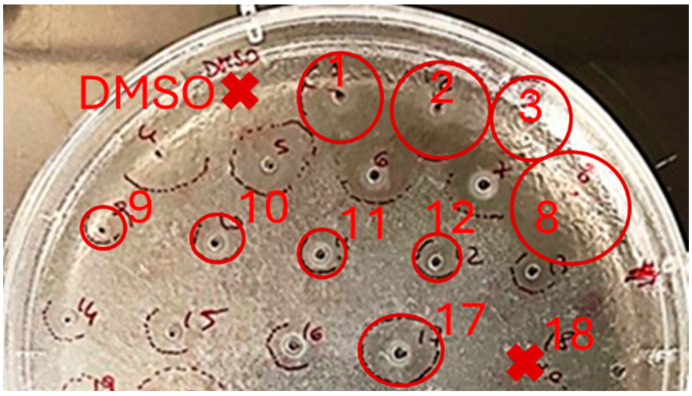
Results of antibacterial activity of plant extracts from the roots and aboveground parts of *L. gmelini* obtained by CM, UE, and SFE against *S. epidermidis* ATCC 12228. Note: Zones of growth inhibition of the test strain are marked by circles. The red cross symbolizes absence of activity.

Aliquots of 3 µL of each sample (10 mg/mL) were applied into holes in the agarose plate. After 24/48 h of incubation, antibacterial activity was evaluated by measuring the diameter of the growth inhibition zones, presented in Figure 4.

The results of the biological screening demonstrated no activity against the reference Gram-negative bacterial strain of *Escherichia coli* ATCC 25922 for all isolated extracts.

At the same time, during comparative screening, it was found that the examined extracts inhibited the growth of the Gram-positive *S. epidermidis* ATCC 12228 bacterial strain (Figure 4), with a higher inhibition observed for the extracts isolated from the roots compared to such from the aboveground parts.

The diameters of the growth inhibition zones for the test strains are presented in Table 3. As internal controls of the experiments, a well-characterized antimicrobial peptide, i.e., Esc(1-21) at 2.2 mg/mL (inhibition zone = 22.6 ± 2.3 mm), and ciprofloxacin at 0.5 mg/mL (inhibition zone = 36.9 ± 1.9 mm) were used.

It was found that the methanolic fractions isolated by SPE from the roots and aboveground parts had inhibition zones of 19.1 ± 1.4 mm and 16.2 ± 02.3 mm, respectively, showing slightly higher activity compared to the total extracts, which had inhibition zones of 13.4 ± 3.7 mm and 8.6 ± 0.2 mm, respectively.

The activity of the aqueous fractions from the roots (inhibition zone 6.3 ± 0.2 mm) was three times lower than that of the methanolic fraction (inhibition zone 19.1 ± 1.4 mm) and twice as low as that of the 1a extract itself (inhibition zone 13.4 ± 3.7 cm). In contrast, the aqueous fractions after SPE from the aboveground parts exhibited no activity at all.

This difference is likely attributed to the enhanced purification of the polyphenolic component in the methanolic fraction, which may increase the antimicrobial activity [36]. These results suggest a possible association between polyphenol enrichment and antibacterial activity, particularly in methanolic SPE fractions, although no statistical correlation was formally performed in this study. Thus, while polysaccharide-rich aqueous extracts showed negligible antimicrobial effects, the polyphenol-enriched methanolic fractions demonstrated significant efficacy against Gram-positive bacteria. The antimicrobial activity of the fractions against *S. epidermidis* ATCC 12228 was also evaluated by measuring the microbial growth in a microdilution broth assay. Inhibitory concentration 50 (IC_50_) and inhibitory concentration 90 (IC^90^) values are reported in Table 4.

It was found that fractions 1c, 2a, 2b, and 2c2 showed IC^90^ values lower than 1 mg/mL, while fractions 1a and 1c2 showed IC^90^ values slightly higher. All the other fractions showed IC^90^ higher than 2 mg/mL. Esc(1–21) (IC^90^ = 3.4 µg/mL) and ciprofloxacin (IC^90^ = 0.12 µg/mL) were used as internal control of the experiments. 

### 2.4. ^1^H-NMR Analysis of Extracts Purified by Solid-Phase Extraction

Due to their significant antimicrobial activity, the methanolic fractions (1c2, 2c2) isolated through SPE were further analyzed using NMR spectroscopy to identify additional bioactive compounds, aside from monosaccharides, that may have been responsible for the observed inhibition of Gram-positive bacteria.

**Figure 5:**
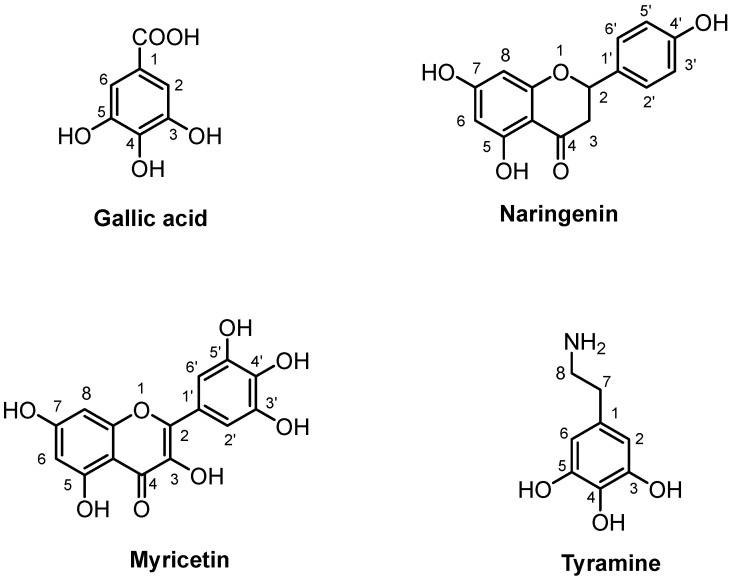
Molecular structures of compounds assigned by ^1^H NMR.

Analysis of the spectra revealed the presence of polyphenols, both in their free forms and as glycosylated compounds, as illustrated in Figure 5 and summarized in Table 5.

Free polyphenols, which are not conjugated with sugar moieties, exhibited typical NMR signals related to aromatic structures and phenolic rings in the down-field region. Conversely, distinctive signals associated with the sugar portion were observed, which may have been bound to the aglycone group through a glycosidic bond. Within the mid-field of the NMR spectrum, signals corresponding to the sugar portion appeared as multiplets related to glucose units, particularly in the chemical shift regions between 3 and 5 ppm. These signals indicated the presence of sugars bound to polyphenols, although the exact hydroxyl group involved in the glycosidic bond could not be determined. The observed spectral patterns indicated that these sugars may have been attached at different positions on the phenolic rings, potentially generating a variety of structural isomers.

Gallic acid, myricetin, and naringenin were identified in the roots and above-ground parts. In addition, tyramine, a biogenic amine derived from lignanamide, was found in plant roots.

The structural assignment of gallic acid was achieved by identifying a singlet at 7.05 ppm in the ^1^H spectrum, corresponding to the protons at positions 2 and 6 of the aromatic ring (Figure 5). Furthermore, the two-dimensional ^1^H-^13^C HSQC experiment elucidated spin correlations between these protons (H-2, H-6) and the carbons at 110.9 ppm. The presence of gallic acid was further corroborated by long-range correlations observed in the two-dimensional ^1^H-^13^C HMBC experiment through the correlation of the protons at 7.05 (H-2, H-6) and the carboxylic group at 170.2 ppm. The amount of gallic acid measured was 543.20 mg and 353.58 mg in the roots and aboveground parts, respectively (Table 5).

The identification of naringenin (Figure 5) was based on detecting the spin systems corresponding to protons H-7 and H-9 at 5.90 and 5.88 ppm, respectively, and the relative coupling *J_meta_* = 2.1. In the phenolic portion, protons H-2′ and H-6′ were observed at 6.80 ppm, showing a correlation in the ^1^H-^1^H TOCSY map with protons H-3′ and H-5′, detected at 7.30 ppm. Additionally, the ^1^H-^13^C HSQC experiment highlighted correlations between the H-2′/H-6′ and H-3′/H-5′ protons with the carbons at 115.0 ppm and 127.8 ppm, respectively. Naringenin quantification showed values of 162.07 mg for the aboveground parts, and 559.70 mg for the roots (Table 5).

The presence of myricetin (Figure 5) was assessed by the observation of the spin systems formed between the protons at 6.37 ppm (H-8, *J* = 2.2) and 6.18 (H-6, *J* = 2.2) of the chromonic moiety in the ^1^H-^1^H TOCSY spectrum. The ^1^H spectrum detected a singlet at 7.30 ppm related to the protons H-2′ and H-6′, which were correlated in the ^1^H-^13^C HSQC to the carbons at 110.3 ppm. The level of myricetin was equal to 507.02 mg in the aboveground parts and 335.56 mg in the roots (Table 4).

The ^1^H NMR spectrum of the radical part also displayed distinct signals of the aromatic portion of tyramine, such as H = 7.21 (H-3, H-5) and H = 6.90 (H-2, H-6), shown in Figure 5. The assignment of the molecule was further confirmed by a coupling *J_orto_* = 8.3 and the correlation of the doublets in the ^1^H-^1^H TOCSY experiment. In addition, the ^1^H spectrum revealed characteristic triplets for the methylene protons at 2.90 (H-7) and 3.22 (H-8), homocorrelated with a *J* = 7.30 and hetero-correlated with C = 30.7 ppm and C = 41.9 ppm, respectively. The amount of tyramine quantified in the roots was 341.34 mg (Table 5).

These compounds are well-documented for their antimicrobial, antioxidant, and anti-inflammatory properties [6,8,36]. Their differential distribution between roots and aerial parts may account for the varied bioactivities observed across extracts. ^1^H NMR analysis of the methanolic fractions revealed a strong presence of polyphenolic compounds, whereas the other fractions were predominantly composed of carbohydrate-rich molecules. This differentiation highlights the effectiveness of SPE in isolating distinct bioactive classes from the complex plant matrix. In particular, the methanolic fractions, characterized by strong signals in the 6–8 ppm range, were rich in polyphenols, confirming the presence of aromatic ring structures (Figure 5). This aligns with the literature on polyphenols in plant extracts, in line with previous studies [33]. The higher antibacterial activity observed in methanolic SPE fractions may be associated with the identified polyphenols, particularly myricetin and naringenin. These compounds have been shown to act through membrane disruption and the induction of oxidative stress in bacterial cells, mechanisms that are well-documented for several flavonoids [37,38,39]. Additionally, the presence of tyramine, possibly related to lignanamide structures characteristic of *L. gmelini* roots [2,5], further contributes to the chemical complexity of the active extracts.

## 3. Materials and Methods

### 3.1. Plant Material

The roots and aboveground parts of *L. gmelini* were collected in the Almaty region during the flowering period of 2020 and identified at the Institute of Botany and Phytointroduction (Almaty, Kazakhstan) under the voucher no. 154958. The collection site coordinates were in the Talgar district, Zhanalyk village (H = 1326 m above the sea level, N = 43°34′28″, E = 077°03′20″).

### 3.2. Sample Preparation

Post-harvest processing involved the removal of dirt and impurities followed by thorough cleaning. The cleaned plant materials were further dried in a well-ventilated, clean area that was shielded from light until a constant weight was achieved, ensuring a weight loss of less than 10% as per the guidelines of the State Pharmacopoeia of the Republic of Kazakhstan.

Subsequently, the dried materials were ground to a 2.0–3.0 mm particle size using a Retsch Cutting Mill SM 200 (Retsch GmbH, Haan, Germany). Quality control was conducted in accordance with the standards set by the State Pharmacopoeia of the Republic of Kazakhstan and the European Pharmacopoeia (8th edition). The ground materials were stored in sealed Kraft paper bags with the plant name, collection site, collection time, and net weight indicated on the labels.

### 3.3. Chemicals and Reagents

Ethanol of ≥90% purity was purchased from “Talgar-Spirits” (Talǧar, Kazakhstan) and used without purification. Luria–Bertani broth (LB, L3022), agarose (A9539), deuterated DMSO ≥99.9% (CAS No. 67-68-5), and Tetramethylsilane (TMS, CAS No. 75-76-3) were purchased from Sigma-Aldrich (St. Louis, MO, USA). Water was purified using a Milli-Q^®^ system from Millipore (Burlington, MA, USA). The antibacterial tests used bacterial strains Gram-negative *E. coli* ATCC 25922 and Gram-positive *S. epidermidis* ATCC 12228, and the IWAKI Tissue Culture Plates (3030-150) were from IWAKI (Tokyo, Japan).

### 3.4. Extraction of Biologically Active Compounds

#### 3.4.1. Ultrasound-Assisted Extraction (UAE)

Ultrasound-assisted extraction (UAE) was performed on crushed plant material (1 and 2) in an ultrasonic bath Elmasonic S 450 (Elma, Singen, Germany) following the method described previously [12]. The filtered hydroalcoholic extract (1a and 2a) was concentrated using a rotary evaporator IKA RV20 (IKA^®^-Werke GmbH & Co., KG, Staufen im Breisgau, Germany) at 40–45 °C under a vacuum until dry, yielding a brown crystalline powder.

#### 3.4.2. Conventional Maceration (CM)

For conventional maceration, the crushed plant material (1 and 2) was subjected to a 5-h double extraction using a six-fold excess of 50% ethanol at 20–23 °C. The raw material was subsequently washed with distilled water to remove any residual substances. The combined extracts (1b and 2b) were concentrated to dryness under a vacuum at 40–45 °C, producing a dry extract [3].

#### 3.4.3. Supercritical Fluid Extraction (SFE)

The CO_2_ supercritical fluid extraction was performed according to Cairone et al., 2023 [36] with some modifications. About 5 g of the roots and aboveground parts (1 and 2) were subjected to CO_2_ supercritical fluid extraction (SFE-CO_2_). The SFE-CO_2_ extraction was performed using a supercritical CO_2_ apparatus provided by Jasco Europe (Cremella, Italy). The system consists of a pump (scCO_2_ Jasco-PU-4347), a controlled temperature extraction oven with a stainless-steel filtering set (Jasco CO-4065), and a back-pressure regulator (Jasco CO_2_-BP-4390). Extraction was thus performed using ethanol as a co-solvent at an optimal ratio of EtOH: H_2_O (75:25, *v*/*v*)/CO_2_ (60:40, *v*/*v*) and a flow rate of 5 mL/min for 4 h at 45 °C and 35 MPa. The resultant ethanolic solutions (1c and 2c) were dried under a vacuum at 40 °C and stored at 4 °C until further analysis.

#### 3.4.4. Solid Phase Extraction (SPE)

The 1c and 2c samples were subjected to SPE using a Discovery^®^DSC-18 SPE Tube column (Merck Life Science, S.r.l., Milan, Italy) to concentrate the polyphenolic fraction [38]. After preconditioning the column with methanol, about 200 mg of the extract was dissolved in methanol and loaded onto the column. The column was sequentially eluted with water to obtain the fractions 1c_1_ and 2c_1,_ and subsequentially eluted with MeOH until the column was fully cleaned to obtain the fraction 1c_2_ and 2_c2_. The fractions were dried under reduced pressure at 30 °C on a rotary evaporator, weighed, and stored at 4 °C until further analysis.

### 3.5. Study of Monosaccharide Composition

Monosaccharides were analyzed after derivatization as AMGs. An amount of 1 mg of sample was dried over P_2_O_5_ for 1 h; after that, the methanolysis was performed in 1 mL of HCl/MeOH 1.25 M at 80 °C for 20 h. The obtained mixture was extracted three times with hexane, and the methanol layer was dried and acetylated with 50 mL of Ac_2_O and 50 mL of Pyr at 100 °C for 30 min. After three extractions with CHCl_3_/H_2_O (*v*:*v*; 1:1), the sample was dissolved in acetone and analyzed by GC-MS. The monosaccharides were identified by Electron ionization (EI) mass spectra and GC retention times by comparison with those of authentic standards.

The linkage positions of the monosaccharides were obtained by the analysis of the partially methylated alditol acetates (PMAAs). The methylation reaction was achieved by incubating 2 mg of each sample with CH_3_I (100 µL) and NaOH powder in 300 µL of dimethyl sulfoxide (DMSO) for 20 h. The product was then hydrolyzed with 2 M trifluoroacetic acid at 120 °C for 2 h, reduced with NaBD_4_, and acetylated.

All the derivatives were analyzed on an Agilent Technologies GC System 7820A (Santa Clara, CA, USA) equipped with a mass selective detector 5977B and an HP-5 capillary column (Agilent, 30 m × 0.25 mm i.d.; flow rate, 1 mL min^−1^, He as carrier gas). AMGs were analyzed using the following temperature program: 40 °C for 3 min, then 140→240 °C at 3 °C min^−1^. The temperature program for PMAAs was the following: 90 °C for 1 min, then 90→140 °C at 25 °C/min, then 140→200 °C at 5 °C/min, then 200→280 °C at 10 °C/min, and finally, 280 °C for 10 min.

### 3.6. In Vitro Antibacterial Activity Study Using Agar Diffusion Method and Microdilution Broth Assay

The inhibition zone assay was performed as previously reported [39]. Bacterial strains were grown at 37 °C in Luria–Bertani broth (LB) with gentle shaking until reaching an optical density (O.D.) of 0.8 at 590 nm. The bacterial culture was diluted 1:2000 and plated on LB-agarose plates. Aliquots of 3 µL of each sample, concentrated to 10 mg/mL in DMSO, were loaded into holes made in the agarose plates. The plates (IWAKI^®^, 3030-150) were incubated for 24–48 h at 37 °C, and the diameters of the inhibition zones were measured using digital caliber. For the microdilution broth assay, the bacteria were grown ad described before and diluted in Mueller–Hinton broth (MH) with serial dilutions of fractions ranging from 500 to 15.6 µg/mL. The final cell concentration was 1 × 10^6^ cells/mL. The plates were incubated at 37° C for 16 h, and microbial growth was evaluated by measuring the O.D. at 590 nm using a microplate reader (Infinite M200; Tecan, Salzburg, Austria). O.D. values were expressed as the percentage of microbial growth relative to the control and used to calculate IC_50_ and IC^90^ values, i.e., the concentration of fractions inhibiting 50% and 90% bacterial growth, respectively.

### 3.7. ^1^H-NMR Analysis

The dried 1c2 and 2c2 extracts (30 mg) were dissolved in a 1.2 mL mixture of 1:5 CD_3_OD/D_2_O (100 mM phosphate buffer, 0.4 mM TSP as an internal standard). NMR analyses were carried out on a JEOL JNM-ECZ 600R spectrometer (JEOL Ltd., Tokyo, Japan) operating at a proton frequency of 600.17 MHz and equipped with a JEOL 5 mm FG/RO DIGITAL AUTOTUNE probe. Spectra processing and signal integration were performed using JEOL Delta software v5.3.1 (JEOL Ltd., Tokyo, Japan). The ^1^H spectra of the samples were recorded using the following parameters: 128 scans, residual water signal suppression with a presaturation pulse, a 7.73 s relaxation delay, a 90° pulse of 11.3 μs, 64 k data points, and a 9000 Hz spectral width. Two-dimensional NMR experiments, including ^1^H-^1^H TOCSY, ^1^H-^1^C HSQC, and ^1^H-^1^H HMBC, were performed, applying the same experimental parameters as for previously investigated plant matrices [40]. Water-soluble metabolites were quantified by integrating the selected signals and normalizing against the TSP methyl group signal (δH = 0.00 ppm), set to 100. Quantification was expressed as mg/100 g ± SD of dry SPE extract (in three replicates).

### 3.8. Statistical Analysis

All experiments were conducted in triplicate, and the results were presented as mean values ± standard deviation. Statistical analysis, including calculation of means and standard deviations, was performed using Microsoft Excel. *p* > 0.05 was considered as statistically significant.

## 4. Conclusions

This comprehensive analysis of *L. gmelini* extracts reveals a complex phytochemical profile, with both polyphenols and polysaccharides contributing to its biological potential. However, the polyphenol-rich fractions, particularly those obtained through methanolic SPE, exhibited the most significant antibacterial activity, particularly against the Gram-positive strain *S. epidermidis*, thereby reinforcing the role of these secondary metabolites as key antimicrobial agents. In contrast, the polysaccharide-rich fractions showed minimal antibacterial activity, despite the structural complexity and branching patterns observed in their monosaccharide composition. While plant-derived polysaccharides from species such as *Astragalus membranaceus* (Fisch.) Bunge and *Salvia* spp. have demonstrated anti-inflammatory effects through NF-κB and MAPK pathways [13,41], and others like those from *Hippophae rhamnoide* L. and *Agrocybe cylindracea* (DC.) Maire possess anti-glycosylation and α-amylase inhibitory effects or antitumor activity [38], their role in the antibacterial activity of *L. gmelini*, as demonstrated in this study, appears negligible. These findings suggest that future research should focus on the selective extraction and characterization of polyphenol-rich fractions, aiming to isolate both known and novel compounds with antimicrobial potential. Enrichment of these compounds may represent a promising strategy for developing natural agents that are effective against multidrug-resistant pathogens [13]. 

Given the chemical profile and general bioactivity of the polyphenol-enriched fractions, *L. gmelini* extracts may represent a promising source of compounds with potential relevance against Gram-positive bacteria. However, further studies aiming to evaluate the antibiofilm potency and using clinically relevant bacterial models—such as *S. aureus* or MRSA—are needed to substantiate their possible application in topical formulations or medical device coatings aimed at preventing biofilm-associated and resistant infections.

Further exploration will focus on the purification and structural elucidation of bioactive compounds from the most active fractions of *L. gmelini* through an integrated analytical approach combining LC-MS, analytical and preparative HPLC, and Supercritical Fluid Chromatography (SFC), enabling the high-resolution separation and targeted quantification of selected metabolites. These efforts will be supported by microbiological assays to confirm the antimicrobial potential of the isolated compounds, thereby laying the groundwork for future in vitro and in vivo investigations aimed at elucidating their mechanisms of action and therapeutic relevance.

Altogether, the current study highlights the pharmacological significance of *L. gmelini* as a valuable source of antimicrobial phytochemicals, promoting further investigation of its properties in the context of natural product-based drug discovery.

## Figures and Tables

**Table 1:** Monosaccharide composition (molar%) of plant extracts from the roots and aboveground parts of *L. gmelini*.

Sample	Rha	Xyl	Ara	GlcA	GalA	Man	Gal	Glc
1a (UAE, roots)	9.7		13.9	<0.5		<0.5	3.0	72.7
1b (CM, roots)	10.1		16.5	<0.5		0.5	3.4	69.3
1c (SFE, roots)	11.3		10.3	<0.5		0.8	2.4	75.1
1c1 (Aqueous fraction from the roots SPE extract)	4.3		1.6	<0.5		0.7	1.6	91.5
2a (UAE, aboveground parts)	10.5	2.0	7.6	2.0	1.1	1.4	11.1	64.3
2b (CM, aboveground parts)	11.0	2.1	6.2	2.0	1.1	1.6	11.5	64.5
2c (SFE, aboveground parts)	10.9	1.6	2.7	1.9	0.7	1.1	7.8	73.3
2c1 (Aqueous fraction from the aboveground parts SPE extract)	4.8	1.4	1.5	0.6	0.4	1.0	3.3	87.0

**Table 2:** Analysis of methylated alditol acetates of fractions 1b and 2b.

Monosaccharide	Xyl	Ara	Rha
Sample			
Root extracts	-	Terminal-Ara	Terminal-Rha 3,4-Rha
Aboveground parts extracts	Terminal-Xyl	Terminal-Ara	3,4-Rha

**Table 3:** Results of the antibacterial activity of plant extracts from the roots and aboveground parts of *L. gmelini* obtained by UAE, CM, and SFE methods against bacterial strains.

No.	Sample	Inhibition Zone (mm)	
		*E. coli* ATCC 25922	*S. epidermidis* ATCC 12228
1	1a (UAE, root)	n.a.	13.4 ± 3.7
2	1b (CM, root)	n.a.	14.3 ± 4.6
3	1c (SFE, root)	n.a.	12.9 ± 3.5
8	1c2 (Methanol fraction from root extract, SPE)	n.a.	19.1 ± 1.4
9	1c1 (Aqueous fraction from root extract, SPE)	n.a.	6.3 ± 0.2
10	2a (UAE, aboveground part)	n.a.	8.6 ± 0.2
11	2b (CM, aboveground part)	n.a.	8.7 ± 1.2
12	2c (SFE, aboveground part)	n.a.	9.3 ± 2.1
17	2c2 (Methanol fraction from aboveground parts extract, SPE)	n.a.	16.2 ± 2.3
18	2c1 (Aqueous fraction from aboveground parts extract, SPE)	n.a.	n.a.
	DMSO (100%)	n.a.	n.a.

n.a. = not active. The data represents the mean ± standard deviation of three independent experiments. Note: Samples numbered 4–7 and 13–16 are also extracts, but they were not included in the comparison or analysis, and for this reason they were not included in Table 3.

**Table 4:** IC_50_ and IC^90^ values (in µg/mL) of different extracts from root and aboveground parts of the plant against *S. epidermidis* ATCC 12228.

No.	Sample	IC_50_ (µg/mL)	IC^90^ (µg/mL)
1	1a (UAE, root)	120.9	1155.3
2	1b (CM, root)	152.4	2481.7
3	1c (SFE, root)	219.2	798.7
8	1c2 (Methanol fraction from root extract, SPE)	101.7	1059.3
9	1c1 (Aqueous fraction from root extract, SPE)	964.3	3797.5
10	2a (UAE, aboveground part)	51.2	162.7
11	2b (CM, aboveground part)	139.3	723.0
12	2c (SFE, aboveground part)	118.2	2195.1
17	2c2 (Methanol fraction from aboveground parts extract, SPE)	101.9	459.1
18	2c1 (Aqueous fraction from aboveground parts extract, SPE)	185.3	3629.3

The data represents the modal value of three independent experiments.

**Table 5:** Metabolites identified in the 600.13 MHz ^1^H NMR spectra of methanolic extracts of the roots parts (1c2) and aboveground parts (2c2), (1:5 CD_3_OD/D_2_O 100 mM phosphate buffer, containing 0.4 mM TSP). The concentration of compounds is expressed as mg/100 g ± SD of dry SPE extract (in three replicates).

Compound	Assignment	^1^H (ppm)	Multiplicity [*J*(Hz)]	^13^C (ppm)	1c2 mg/100g	2c2 mg/100g
Gallic Acid	CH-2, CH-6	7.05 *	s	110.9	543.20 ± 0.05	353.58 ± 0.04
	COOH			170.2		
Myricetin	CH-8	6.37 *	d [2.2]	95.0	507.02 ± 0.07	335.56 ± 0.10
	CH-6	6.18	d [2.2]	100.0		
	CH-2′, CH-6′	7.30	s	110.3		
Naringenin	CH-3a, CH-3b	2.68, 3.10	dd, dd [17.0; 3.0]	42.7	162.07 ± 0.09	559.70 ± 0.04
	CH-2	5.30	dd [12.6, 2.7]	79.0		
	CH-6	5.88	d [2.1]	95.7		
	CH-8	5.90 *	d [2.1]	94.9		
	CH-2′, CH-6′	6.80	m	115.0		
	CH-3′, CH-5′	7.30	m	127.8		
Tyramine	CH_2_-7	2.90	t [7.3]	30.7	n.d.	341.34 ± 0.05
	CH_2_-8	3.22	t [7.3]	41.9		
	CH-2, CH-6	6.90 *	d [8.3]	115.5		
	CH-3, CH-5	7.21	d [8.3]	131.3		

The exponents (*) indicate signals selected for integration.

## Data Availability

All data is contained within the article.
